# Prevalence, correlates and in-hospital outcomes of kidney dysfunction in hospitalized patients with heart failure in Buea-Cameroon

**DOI:** 10.1186/s12882-021-02641-2

**Published:** 2022-01-03

**Authors:** Ahmadou Musa Jingi, Clovis Nkoke, Jean Jacques Noubiap, Denis Teuwafeu, Alex T. Mambap, Cyrille Nkouonlack, Ronald Gobina, Debimeh Njume, Anastase Dzudie, Gloria Ashuntantang

**Affiliations:** 1 Faculty of Health Sciences, University of Bamenda, Bamenda, Cameroon; 2 Buea Regional Hospital, Buea, Cameroon; 3 Clinical Research Education, Networking and Consultancy (CRENC), Douala, Cameroon; 4 Center for Heart Rhythm Disorders, University of Adelaide, Adelaide, SA Australia; 5 Faculty of Health Sciences, University of Buea, Buea, Cameroon; 6 Faculty of Medicine and Biomedical Sciences, University of Yaounde 1, Yaoundé, Cameroon

**Keywords:** Kidney dysfunction, Heart failure, Outcome, Mortality, Cameroon, Sub-Saharan Africa

## Abstract

**Background:**

Kidney dysfunction is common in patients with heart failure (HF) and has been associated with poor outcomes. This study aimed to determine the prevalence, correlates, and prognosis of kidney dysfunction in patients with HF in Cameroon, an understudied population.

**Methods:**

We conducted a cross-sectional study in consecutive patients hospitalized with HF between June 2016 and November 2017 in the Buea Regional Hospital, Cameroon. Kidney dysfunction was defined as an estimated glomerular filtration rate < 60 ml/min/1.73m^2^. Prognostic outcomes included death and prolonged hospital stay (> 7 days). We also performed a sensitivity analysis excluding racial considerations.

**Results:**

Seventy four patients (86.1% of those eligible) were included. Their median age was 60 (interquartile range: 44–72) years and 46.0% (*n* = 34) were males. Half of patients (*n* = 37) had kidney dysfunction. Correlates of kidney dysfunction included previous diagnosis of HF (adjusted odds ratio [aOR]4.3, 95% CI: 1.1–17.5) and left ventricular hypertrophy (aOR3.4, 95% CI: 1.1–9.9). Thirty-six (48.9%) had prolonged hospital stay, and seven (9.5%) patients died in hospital. Kidney dysfunction was not associated with in-hospital death (aOR 0.4, 95% CI: 0.1–2) nor prolonged hospital stay (aOR 2.04, 0.8–5.3). In sensitivity analysis (excluding racial consideration), factors associated with Kidney dysfunction in HF were; anemia (aOR: 3.0, 95% CI: 1.1–8.5), chronic heart failure (aOR: 4.7, 95% CI: 0.9–24.6), heart rate on admission < 90 bpm (aOR: 3.4, 95% CI: 1.1–9.1), left atrial dilation (aOR: 3.2, 95% CI: 1.04–10), and hypertensive heart disease (aOR: 3.1, 95% CI: 1.2–8.4). Kidney dysfunction in HF was associated with hospital stay > 7 days (OR: 2.6, 95% CI: 1–6.8).

**Conclusion:**

Moderate-to-severe kidney dysfunction was seen in half of the patients hospitalized with HF in our setting, and this was associated with a previous diagnosis of HF and left ventricular hypertrophy. Kidney dysfunction might not be the main driver of poor HF outcomes in this population. In sensitivity analysis, this was associated with anemia, chronic heart failure, heart rate on admission less than 90 bpm, left atrial dilatation, and hypertensive heart disease. Kidney dysfunction was associated with hospital stay > 7 days.

**Supplementary Information:**

The online version contains supplementary material available at 10.1186/s12882-021-02641-2.

## Introduction

Heart failure and chronic kidney diseases (CKDs) are common conditions in sub-Saharan Africa (SSA), where they account for about 35% of all deaths [1–3]. Heart failure and CKD share common risk factors such as hypertension, obesity, and diabetes that have high prevalence rates in the region [1, 4–7]. In Cameroon, about one in three adults is hypertensive [8, 9] and one in nine has diabetes [10]. The burden of HF in the community is not known in our setting. Heart failure accounts for about 5% of hospital consultations and admissions in adults, and hypertensive heart disease is the predominant cause of HF [4–6].

The community prevalence of CKD ranged from 10 to 14% [11]. This varied between risk groups and can be as high as 52% in those with hypertension [11, 12]. Kidney dysfunction and heart failure frequently co-exist and interact in a complex bi-directional and interdependent manner known as a cardiorenal syndrome [13]. Moderate-to-severe kidney dysfunction (eGFR < 60 ml/min/1.73m^2^) can be seen in more than 30% of patients with heart failure [14, 15] and has been associated with increased mortality [15, 16]. There is a dearth of data on the burden of kidney dysfunction in patients with heart failure in Cameroon (SSA), especially in semi-urban settings. This study aimed to determine the prevalence, correlates, and prognosis (in-hospital death and prolonged hospital stay) of kidney dysfunction in patients with heart failure admitted in a semi-urban setting in Cameroon.

## Methods

### Study design and setting

We carried out a prospective cross-sectional study in patients hospitalized with HF between June 2016 and November 2017 in the Internal Medicine Unit of the Buea Regional Hospital in the South West region of Cameroon. This is a second-level hospital with a bed capacity of 111 and a catchment population of over 200,000 inhabitants. The hospital serves as a teaching hospital for the Faculty of Health Sciences of the University of Buea and is equipped and staffed with personnel who are trained to diagnose and treat heart and kidney diseases. At the time of the study, CN was the only cardiologist in the region and who made the diagnosis of HF in all the patients.

### Study population and sample size

We included all consenting patients aged ≥18 years who were hospitalized with HF, with kidney function assessed on admission. The patients were consecutively recruited into an HF registry.

### Data collection

The methods involved in this study have been previously described [5]. The data were prospectively collected with a predefined questionnaire. This constituted the first HF registry in the region. We collected demographic data (age and sex), medical history (previous diagnosis of HF, hypertension, diabetes, atrial fibrillation, alcohol consumption, tobacco use). Each patient underwent a complete clinical evaluation for symptoms and signs of HF (CN). We measured the blood pressure according to standard procedures and a blood sample was collected for biochemical analysis including serum creatinine, sodium, potassium, hemoglobin, and fasting blood glucose. Each patient underwent a 12-lead resting electrocardiogram and a comprehensive cardiac ultrasound by a cardiologist (CN) using a SonoscapeS8 ultrasound machine (Sonoscape, China).

### Variables

Moderate-to-severe kidney dysfunction was defined as an estimated glomerular filtration rate (eGFR) < 60 ml/min/1.73m^2^), similar to that reported in the European Society of Cardiology guidelines on heart failure and based on the Kidney Disease Outcome Quality Initiative (KDOQI) guidelines [17, 18]. The eGFR (in mL/min.1.73m^2^) was estimated using the simplified Modification of Diet in Renal Disease (MDRD) equation: (186.3 × (serum creatinine (mg/dl))–1.154 × age–0.203 × (0.742 if female) × (1.212 if black African). Those with any kidney impairment was defined as an eGFR < 90 ml/min/1.73m^2^. The severity of kidney impairment was defined according to the Kidney Disease Outcome Quality Initiative (KDOQI) as mild (60 – 89 ml/min/1.73m^2^), moderate (30–59 ml/min/1.73m^2^), severe (15 – 29 ml/min/1.73m^2^), and failure (< 15 ml/min/1.73m^2^, 19]. We considered hyper-filtration as an eGFR > 130 ml/min/1.73m^2^ [19]. Outcome variables were in-hospital death or prolonged hospital stay (> 7 days).

### Statistical analyses

We analyzed the data using IBM SPSS version 26 (IBM Corp, Armonk, NY, USA). Continuous variables are presented as means with standard deviation (SD) or median with interquartile range (IQR), and discrete variables as frequencies and proportions with the corresponding 95% confidence intervals (95% CI). We compared means with independent sample T-test and proportions with Chi-squared or Fischer exact test where appropriate. We assessed for factors associated with an eGFR < 60 ml/min/1.73m^2^ in patients with HF in bivariate analyses and then adjusted for age (with a 55 years cutoff). We then assessed the association between eGFR and outcome measures of death or prolonged hospitalization using multivariable regression analysis with adjustment for age (55 years cutoff) and sex. We also conducted a sensitivity analysis with an eGFR < 60 ml/min/1.73m^2^ without considering the black race. There were no missing data for the relevant variables studied. A *p*-value < 0.05 was considered statistically significant for observed differences or associations.

### Ethical considerations

This study was approved by the administrative authority of the Buea Regional Hospital acting as the local ethics committee. All participants or their legal guardians provided written informed consent to be included in the heart failure registry. All the patients approached accepted to be included in the registry.

## Results

### General characteristics

During the study period, 86 consecutive patients were hospitalized with HF. Of these, 74(86.1%) patients had serum creatinine data on admission and were therefore included. The clinical characteristics are shown in Table 1. Thirty-four patients (46.0%) were males. Their age ranged from 22 to 100 years, with a median (IQR) of 60 (IQR: 44–72) years (Table 1). Hypertension was the most frequent cardiovascular risk factor, seen in 42 (56.8%, [95% CI: 44.7–68.2]) patients. Previous diagnosis of HF was reported by 13 (17.6%, [95% CI: 9.7–28.2]) patients. These patients with known HF were all on diuretics (non-anti-aldosterone), eight on angiotensin-converting enzyme inhibitors, five on beta-blockers, and six on spironolactone. Five patients (6.8%, [95% CI: 2.2–15.1]) had a history of CKD. The echocardiographic and biochemical characteristics are shown in Table 2. Left atrial dilatation was the most frequent echocardiographic finding, seen in 58 (78.1%, [95% CI: 67.3–87.1]) patients. Biventricular heart failure was the most frequent type, seen in 42 (56.8%, [95% CI: 44.7–68.2]) patients. Hypertensive heart failure was the most frequent etiology of HF.

**Table 1:** Clinical characteristics of the participants hospitalized with heart failure (n = 74): overall and by categories of kidney function

Variable	Overall (*n* = 74)	eGFR (ml/min/1.73m^2^)		*p-*value
		< 60 (*n* = 37)	≥ 60 (*n* = 37)	
Age (years), mean (SD)	58.7 (17.9)	61.9 (16.6)	55.5 (18.9)	0.4
Male sex, n(%)	34 (45.9)	17 (45.9)	17 (45.9)	> 0.9
**Medical History, n(%)**				
Chronic Heart Failure	13 (17.6)	10 (27)	3 (8.1)	0.03
Hypertension	42 (56.8)	22 (59.5)	20 (54.1)	0.6
Diabetes	11 (14.9)	5 (13.5)	6 (16.2)	0.7
Current smoking	6 (8.1)	4 (10.8)	2 (5.4)	0.4
Atrial Fibrillation	2 (2.7)	2 (5.4)	0 (0)	0.2
Chronic Kidney Disease	5 (6.8)	5 (13.5)	0 (0)	0.02
Alcohol consumption	9 (12.2)	5 (13.5)	4 (10.8)	0.7
**Symptoms, n (%)**				
NYHA Class				
II	5 (6.8)	1 (2.7)	4 (10.8)	0.2
III	38 (51.4)	23 (62.2)	15 (40.5)	
IV	31 (41.9)	13 (35.1)	18 (48.6)	
Fatigue	74 (100)	37 (100)	37 (100)	NA
Orthopnea	70 (94.6)	36 (97.3)	34 (91.9)	0.3
**Physical findings**				
Systolic BP, mean (SD), mmHg	142.7 (36)	146.8 (39.7)	138.6 (31.8)	0.3
Diastolic BP, mean (SD),mmHg	94.2 (28.5)	97.7 (34.4)	90.8 (20.8)	0.2
Mean BP, mean (SD), mmHg	110.4 (29.6)	114.1 (34.7)	106.7 (23.4)	0.4
Heart Rate, mean (SD), bpm	96.5 (20.2)	94.3 (24.6)	98.6 (14.5)	0.02
Pedaledema, n(%)	66 (89.2)	35 (94.6)	31 (83.8)	0.2
Rales, n (%)	58 (78.4)	30 (81.1)	28 (75.7)	0.6
**Etiologies of Heart Failure**				
Rheumatic Heart Disease	5 (6.8)	3 (8.1)	2 (5.4)	0.6
Hypertensive Heart Disease	36 (48.7)	22 (59.5)	14 (37.8)	0.06
Idiopathic dilatedCardiomyopathy	7 (9.5)	5 (13.5)	2 (5.4)	0.2
Ischemic Heart Disease	8 (10.8)	1 (2.7)	7 (18.9)	0.03
HIV Heart Disease	1 (1.4)	0	1 (2.7)	NA
Cor Pulmonale	8 (10.8)	2 (5.4)	6 (16.2)	0.13
Peripartum Cardiomyopathy	1 (1.4)	1 (2.7)	0	NA
Others	8 (10.8)	3 (8.1)	5 (13.5)	0.46

*BP* Blood Pressure; *eGFR* estimated Glomerular Filtration Rate; *NA* non-applicable, *NYHA* New York Heart Association; *SD* Standard Deviation

**Table 2:** Echocardiographic and biochemical characteristics in patients hospitalized with heart failure (*n* = 74): overall and by categories of kidney function

Variable	Overall (*n* = 74)	eGFR (ml/min/1.73m^2^)		*p-*value
		< 60 (*n* = 37)	≥60 (*n* = 37)	
**Mean (SD) values**				
Septum (mm)	10.3 (2.8)	10.9 (3.1)	9.8 (2.4)	0.12
Posterior wall (mm)	10.4 (2.7)	10.9 (3.2)	9.8 (1.9)	< 0.01
LV End-diastolic diameter (mm)	57.8 (11)	59.5 (8.9)	48.8 (11.8)	0.03
LV End-systolic diameter (mm)	46.7 (13.4)	48.8 (11.8)	44.5 (14.7)	0.1
Relative Wall Thickness	0.38 (0.2)	0.38 (0.13)	0.38 (0.17)	0.4
LV Mass (g)	249.4 (101.4)	278.8 (111)	219 (81.4)	0.1
LV Ejection fraction (%)	38.7 (19.6)	37.2 (16.1)	40.2 (22.7)	< 0.01
LV Fractional shortening (%)	19.7 (11.9)	18.4 (9.8)	21.1 (13.7)	0.01
LA area (cm^2^)	24.7 (6.9)	25.4 (5.9)	24 (7.7)	0.2
LA Diameter (mm)	41.6 (8.4)	43.2 (8)	40.1 (8.7)	0.5
RA Area (cm^2^)	20.7 (6.9)	20.3 (6.1)	21.1 (7.7)	0.5
TAPSE (mm)	15.3 (2.3)	15.5 (2.4)	15 (2.2)	0.7
E-wave Deceleration time (ms)	118.2 (36.1)	120.6 (35.3)	115.9 (37.2)	0.7
E/A ratio	1.88 (0.95)	1.82 (0.8)	1.93 (1.04)	0.6
PASP (mmHg)	63.7 (20.5)	62.1 (19)	65.6 (22.3)	0.3
Hemoglobin (g/dL)	12.3 (2.4)	11.97 (2.2)	12.7 (2.6)	0.5
Creatinine (mg/L)	18.4 (20.5)	26.6 (26.4)	10.1 (3.9)	< 0.01
eGFR (ml/min/1.73m^2^)	68.98 (20.5)	39.99 (15.9)	97.99 (28.8)	< 0.01
Natriemia (mmol/L)	138.8 (8.3)	138.5 (9.1)	139.1 (7.3)	0.8
Kaliemia (mmol/L)	3.9 (0.8)	3.96 (0.8)	3.86 (0.8)	0.7
Glycemia (g/L)	1.22 (0.6)	1.16 (0.4)	1.29 (0.8)	0.05
**Proportion, n(%) values**				
LV hypertrophy	53 (71.6)	31 (83.8)	22 (59.6)	0.02
LA dilation	58 (78.4)	31 (86.5)	26 (70.3)	0.1
RA dilation	33 (44.6)	21 (56.8)	19 (51.4)	0.6
Type of HF syndrome				
Isolated left Ventricular Failure	22 (29.7)	13 (35.1)	9 (24.3)	0.3
Isolated right Ventricular Failure	10 (13.5)	3 (8.1)	7 (18.1)	
Bi-ventricular Failure	42 (56.8)	21 (56.8)	21 (56.8)	
LV Ejection Fraction < 40%	43 (58.1)	23 (62.2)	20 (54.1)	0.5
TAPSE < 17 mm	52 (70.3)	24 (64.9)	28 (75.7)	0.3
PASP > 35 mmHg	53 (71.6)	27 (73)	26 (70.3)	0.8
Anemia	29 (39.2)	17 (45.9)	12 (32.4)	0.2

*eGFR* estimated glomerular filtration rate; *HF* Heart Failure. *LA* Left Atrium. *LV* Left Ventricle; *PASP* Pulmonary Artery Systolic Pressure; *MDRD* Modifying Diet in Renal Disease; *RA* Right Atrium, *TAPSE* Tricuspid Annular Plane Systolic Excursion

### Frequency of kidney dysfunction

**Fig. 1:**
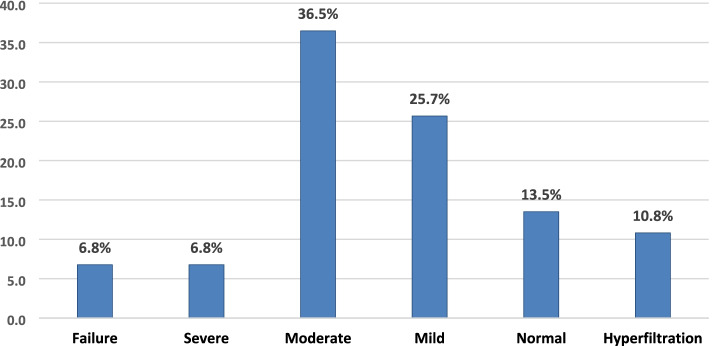
Distribution of the severity of renal dysfunction in patients admitted for heart failure(*n* = 74)

The eGFR ranged between 4.2 and 161.2 ml/min/1.73 m2, with a median of 60.3 (IQR44–88.9). There was no statistically significant difference in eGFR between males (64.5 [SD 33.7]) and females (72.8 [SD 40.0]), *p* = 0.614. Any kidney dysfunction (eGFR < 90 ml/min/1.73m^2^) was seen in 56 (75.7%, [95% CI: 64.3–84.9]) patients. Moderate-to-severe kidney dysfunction (eGFR < 60 ml/min/1.73m^2^) was seen in 37 (50%, [95% CI: 38.1–61.9]) patients. Most of the patients with HF had mild kidney dysfunction (Fig. 1). All cases of kidney dysfunction were seen in those with left ventricular failure. Patients with a history of HF were more likely to have kidney impairment (Table 1). Those with kidney impairment significantly had lower heart rates, lower ejection fraction, and left ventricular hypertrophy (Tables 2 and 3). After adjusting for age > 55 years, a history of chronic heart failure and increased LV mass parameters on echocardiography was associated with moderate to severe kidney impairment (Table 3).

**Table 3:** Factors associated with an eGFR < 60 ml/min/1.73m^2^(n = 37) in patients hospitalized with heart failure (n = 74) (bivariate analyses and adjusting for age > 55 years)

Variable	N (%)	Unadjusted		Adjusted	
		OR (95% CI)	*p*-value	aOR (95%CI)	*p*-value
**Clinical data**					
Age > 55 years					
Yes	25 (52.1)	1.3 (0.5–3.3)	0.6	NA	
No	12 (46.2)	1			
History of Diabetes mellitus					
Yes	5 (45.5)	0.8 (0.2–2.9)	0.7	NA	
No	32 (50.8)	1			
History of Hypertension					
Yes	22 (52.4)	1.3 (0.5–3.1)	0.6	NA	
No	15 (46.9)	1			
Smoking					
Yes	4 (66.7)	2.1 (0.4–12.4)	0.7	NA	
No	33 (48.5)	1			
Alcohol					
Yes	5 (55.6)	1.3 (0.3–5.2)	0.7	NA	
No	32 (49.2)	1			
Anemia					
Yes	17 (58.6)	1.8 (0.7–4.6)	0.2	1.76 (0.7–4.6)	0.4
No	20 (44.4)	1		1	
Chronic Heart Failure					
Yes	10 (76.9)	4.2 (1.1–16.8)	0.03	4.3 (1.1–17.5)	0.03
No	27 (44.3)	1		1	
NYHA class 4					
Yes	13 (41.9)	0.6 (0.2–1.5)	0.2	0.6 (0.2–1.5)	0.3
No	24 (55.8)	1		1	
Heart rate > 90/min					
Yes	19 (40.4)	0.3 (0.1 6 0.9)	0.03	0.4 (0.1–0.9)	0.04
No	18 (66.7)	1		1	
Mean BP < 80 mmHg					
Yes	4 (66.7)	2.1 (0.4–12.4)	0.7	NA	
No	33 (48.5)	1			
Pedal edema					
Yes	35 (53)	3.4 (0.6–18)	0.3	3.3 (0.6–17.5)	0.3
No	2 (25)	1		1	
Rales					
Yes	30 (51.7)	1.4 (0.5–4.2)	0.8	NA	
No	7 (43.8)	1			
**Echocardiography**					
Left Ventricular Hypertrophy					
Yes	31 (58.5)	3.5 (1.2–10.5)	0.02	3.4 (1.1–9.9)	0.02
No	6 (28.6)	1		1	
LVEF < 40%					
Yes	23 (53.5)	1.4 (0.6–3.2)	0.5	NA	
No	14 (45.2)	1			
Left Atrial Dilation					
Yes	32 (55.2)	2.7 (0.8–8.8)	0.1	2.6 (0.8–8.1)	0.1
No	5 (31.3)	1		1	
Left Heart failure					
Yes	34 (52.3)	2.2 (0.5–9.5)	0.5	NA	
No	3 (33.3)	1			
**Etiology of Heart Failure**					
Hypertensive Heart Disease					
Yes	22 (61.1)	2.4 (0.95–6.1)	0.06	2.35 (0.92–6)	0.07
No	15 (39.5)	1		1	
Ischemic Heart Disease					
Yes	1 (12.5)	0.12 (0.01–1.02)	0.03	0.1 (0.01–0.9)	0.02
No	36 (54.6)	1		1	
Dilated Cardiomyopathy					
Yes	5 (71.4)	2.7 (0.5–15.1)	0.2	NA	
No	32 (47.8)	1			
Cor pulmonale					
Yes	2 (25)	0.3 (0.1–1.6)	0.1	NA	
No	35 (53)	1			
Rheumatic Heart Disease					
Yes	3 (60)	1.5 (0.2–9.8)	0.6	NA	
No	34 (49.3)	1			

*aOR* adjusted Odds Ratio, *BP* blood Pressure, *CI* Confidence Interval, *LVEF* Left Ventricular Ejection Fraction, *NA* Not computed, *NYHA* New York Heart Association

### Outcomes

Patients spent between 3 and 21 days in the hospital, with a median of 7.5 (IQR 6–11) days. Thirty-six (48.9%, [95% CI: 36.9–60.6]) patients had prolonged hospital stay (> 7 days). Kidney dysfunction was not significantly associated with prolonged hospital stay after adjusting for sex and age (adjusted odds ratio [aOR] 2.04, 95% CI: 0.8–5.3).

In-hospital death occurred in seven (9.5%, [95% CI: 3.5–18.5]) patients, including two with hyper-filtration, three with mild kidney dysfunction, and two with moderate kidney dysfunction. No patient with severe kidney dysfunction or those with normal kidney function died. Kidney dysfunction was not associated with in-hospital death (aOR 0.4, 95% CI: 0.1–2). Those with kidney hyper-filtration (compared with those without) had a 4-fold higher in-hospital death (25% versus 7.6%; OR: 4.1, 95% CI: 0.7–25.7]). This 4-fold higher odds for in-hospital death persisted after adjusting for sex and age (aOR 4.3, 95%CI: 0.63–23).

.

### Sensitivity analysis

After re-calculating the eGFR and excluding racial consideration, the median eGFR was 46.8 (IQR: 35–73.3) ml/min/1.73m^2^. This was significantly lower than when the race was considered (mean difference of 12.7 ml/min/1.73m^2^, *p* < 0.001). The number of patients with an eGFR < 60 was 45 (60.8%, [95% CI: 48.8–72]) thus, a re-classification of 8 (10.8%) patients to lower eGFR class (Kappa: 0.784, p < 0.001). The eGFR was normal in 12 (16.2%), mildly reduced in 16 (21.6%), moderately reduced in 35 (47.3%), severely reduced in 4 (5.4%), and failure in 6 (8.1%). Hyper-filtration was seen in 1 (1.4%) patient. Factors associated with kidney dysfunction (Table S1) were; anemia (aOR: 3.0, *p* = 0.036), chronic heart failure (aOR: 4.7, *p* = 0.052), heart rate at admission < 90 bpm (aOR: 3.4, *p* = 0.032), left atrial dilation (aOR: 3.2, *p* = 0.034), and hypertensive heart disease (aOR: 3.1, *p* = 0.021). Kidney dysfunction in HF was associated with hospital stay > 7 days (OR: 2.6, [95% CI: 1–6.8], *p* = 0.050) and not with in-hospital death (OR: 0.9, [95% CI: 0.2–4.1], *p* = 1.00).

## Discussion

This study aimed to investigate the frequency, associated factors, and outcomes of kidney dysfunction in patients hospitalized for HF in a semi-urban setting in Cameroon. We found that moderate-to-severe kidney dysfunction (assessed with the MDRD equation considering black race) was seen in half of the patients hospitalized with HF and this was associated with a previous diagnosis of HF and left ventricular hypertrophy. In the sensitivity analysis (excluding racial consideration), kidney dysfunction was seen in over 60 % of patients admitted with HF and this was associated with anemia, left atrial dilatation, and hypertensive heart disease. Kidney dysfunction might not be associated with in-hospital death but with prolonged hospital stay> 7 days.

Data on the prevalence and correlates of kidney dysfunction in patients with HF in Cameroon are scarce [3]. The prevalence of kidney dysfunction in this study was significantly higher than that reported by other authors. In The Sub-Saharan Africa Survey of Heart Failure (THESUS–HF), 30.6% of patients admitted with acute HF had kidney dysfunction [15]. In a multicenter study of patients with HF in SSA, *Sani* et al. reported a prevalence of 30.6% of kidney dysfunction on admission despite comparable aetiologies of HF with our study (predominant hypertensive heart disease). This could be due to the older age of our patients (60 vs 52 years), as kidney function declines with age [11]. In the CHARM study, *Hillege* et al. reported a prevalence of 36% in an older population of patients with HF with predominantly ischemic heart disease [14]. In a meta-analysis involving over 80 thousand patients, moderate-to-severe kidney impairment was reported in 29% of patients with HF [16]. In a group of patients in Cameroon with hypertension and an average age of 60 years, *Kaze* et al. [12] reported a similar prevalence of about 50%. In another group of patients with hypertension in Cameroon and an average age of 52 years, *Hamadou* et al. [20] reported a prevalence of 33%, similar to that reported by *Sani* et al. [15]. This suggests that in SSA patients (similar age) with high rates of hypertension and kidney dysfunction, the occurrence of HF did not increase the rate of kidney dysfunction.

Factors traditionally associated with CKD in the general population are age, hypertension, diabetes, and obesity [11, 20, 21]. However, the determinants of kidney dysfunction in this small cohort were a history of HF and left ventricular hypertrophy. Those with kidney dysfunction also had statistically non-significant higher odd of the classic risk factors for CKD, with 2 fold odds associated with hypertension and smoking, and 1.6 fold odds associated with age. The relatively small sample did not permit us to detect statistically significant associations. The excess risk of kidney dysfunction in those with a history of HF could be explained by the treatment of HF– the use of diuretics and Angiotensin Converting Enzyme inhibitors or Angiotensin Receptor Blockers. Left ventricular hypertrophy is highly prevalent in patients with CKD, where it constitutes an independent risk factor for arrhythmias, sudden death, and progression of HF [22].

Kidney dysfunction is associated with excess mid- to-long-term mortality in patients with HF [16]. In this study, kidney dysfunction was not associated with in-hospital death. We did not capture follow-up data after discharge. We found high but not significant odds of in-hospital death in patients with HF and renal hyperfiltration. Renal hyperfiltration is associated with increased all-cause mortality in the general population, patients with diabetes, and those with atherosclerotic vascular disease [23–25]. The possible association between renal hyperfiltration and in-hospital death could be due to inherent adverse kidney disorder or marker of sarcopenia–associated with poor outcome in HF.

This study has some limitations. The accuracy of our estimates is limited by the relatively small sample size of the study, as shown by wide confidence intervals. The low number of patients in regression analyses might have precluded the detection of some significant associations. Furthermore, we did not collect data on proteinuria, an important marker of kidney dysfunction. In the absence of available kidney function before the index admission, it was not possible to differentiate CKD from acute kidney injury. Also, it was not possible to tell if the patients with high eGFR were true cases of glomerular hyperfiltration or due to sarcopenia with resultant low serum creatinine levels. There was no follow-up data to assess for medium and long-term outcomes. Despite these limitations, to the best of our knowledge, this study is the first to investigate the epidemiology of kidney dysfunction in patients with HF in a non-urban setting in Cameroon.

## Conclusion

Moderate-to-severe kidney dysfunction (assessed with the MDRD equation and considering the black race) was seen in half of the patients hospitalized with HF in this setting, and this was associated with a previous diagnosis of heart failure and left ventricular hypertrophy on echocardiography. Kidney dysfunction was not associated with in-hospital death or hospital stay > 7 days with the index hospitalization. We also found that patients with high eGFR were more likely to die during admission. Excluding racial consideration in the estimate (sensitivity analysis), mild-to-moderate kidney dysfunction was seen in 60 % of patients admitted with HF, and this was associated with anemia, chronic heart failure, heart rate at admission less than 90 bpm, left atrial dilatation, and hypertensive heart disease. Kidney dysfunction was associated with hospital stay > 7 days.

There is a need for further studies with a larger cohort of patients with appropriate follow-up to better characterize the association of renal dysfunction with heart failure outcome in our context.

## Supplementary Information

**Additional file 1.**

## Data Availability

The datasets used and/or analyzed during the current study are available from the corresponding author on reasonable request.
