# Supplementary material for: Prevalence, correlates and in-hospital outcomes of kidney dysfunction in hospitalized patients with heart failure in Buea-Cameroon
Source: BMC Nephrol. 2022 Jan 3;23:8. doi: 10.1186/s12882-021-02641-2 (PMC8722319; doi:10.1186/s12882-021-02641-2)
Supplement: Supplementary file 1 — Additional file 1. [file 12882_2021_2641_MOESM1_ESM.docx]

Suppl Table 1: Sensitivity analysis of factors associated with an eGFR <60ml/min/1.73m^2^ in patients hospitalized with heart failure (n=74) (bivariate analyses and adjusting for age> 55 years).

| Variable | N (%) | Unadjusted | |  | Adjusted | |
| --- | --- | --- | --- | --- | --- | --- |
|  |  | OR (95% CI) | *p*-value |  | aOR (95%CI) | *p*-value |
| **Clinical data** |  |  |  |  |  |  |
| Age > 55 years |  |  |  |  |  |  |
| Yes | 31 (64.6) | 1.6 (0.6 – 4.1) | 0.4 |  | NA |  |
| No | 14 (53.9) | 1 |  |  |  |  |
| Male sex |  |  |  |  |  |  |
| Yes | 22 (64.7) | 1.4 (0.5 – 3.5) | 0.5 |  | NA |  |
| No | 23 (57.5) | 1 |  |  |  |  |
| History of Diabetes mellitus |  |  |  |  |  |  |
| Yes | 7 (63.6) | 1.2 (0.3 – 4.3) | 0.8 |  | NA |  |
| No | 38 (60.3) | 1 |  |  |  |  |
| History of Hypertension |  |  |  |  |  |  |
| Yes | 29 (69.1) | 2.2 (0.9 – 5.8) | 0.1 |  | 2.1 (0.8 – 5.8) | 0.2 |
| No | 16 (50) | 1 |  |  | 1 |  |
| Smoking |  |  |  |  |  |  |
| Yes | 4 (66.7) | 1.3 (0.2 – 7.7) | 0.8 |  | NA |  |
| No | 41 (60.3) | 1 |  |  |  |  |
| Alcohol |  |  |  |  |  |  |
| Yes | 6 (66.8) | 1.3 (0.3 – 5.8) | 0.7 |  | NA |  |
| No | 39 (60) | 1 |  |  |  |  |
| Anemia |  |  |  |  |  |  |
| Yes | 22 (75.9) | 3.01 (1.1 – 8.4) | 0.03 |  | 3 (1.1 – 8.5) | 0.04 |
| No | 23 (51.1) | 1 |  |  | 1 |  |
| Chronic Heart Failure |  |  |  |  |  |  |
| Yes | 11 (84.6) | 4.4 (0.9 – 21.4) | 0.05 |  | 4.7 (0.9 – 24.6) | 0.05 |
| No | 34 (55.7) | 1 |  |  | 1 |  |
| NYHA class 4 |  |  |  |  |  |  |
| Yes | 19 (61.3) | 1.04 (0.4 6 2.7) | 0.9 |  | NA |  |
| No | 26 (60.5) | 1 |  |  |  |  |
| Heart rate < 90/min |  |  |  |  |  |  |
| Yes | 21 (77.8) | 3.4 (1.2 – 9.8) | 0.02 |  | 3.4 (1.1 – 9.1) | 0.03 |
| No | 24 (51.1) | 1 |  |  | 1 |  |
| Mean BP <80 mmHg |  |  |  |  |  |  |
| Yes | 4 (66.7) | 1.3 (0.2 – 7.7) | 0.8 |  | NA |  |
| No | 41 (60.3) | 1 |  |  |  |  |
| Pedal edema |  |  |  |  |  |  |
| Yes | 42 (63.6) | 2.9 (0.6 – 13.3) | 0.2 |  | 2.9 (0.6 – 13.3) | 0.2 |
| No | 3 (37.5) | 1 |  |  | 1 |  |
| Rales |  |  |  |  |  |  |
| Yes | 37 (63.8) | 1.8 (0.6 – 5.4) | 0.3 |  | NA |  |
| No | 8 (50) | 1 |  |  |  |  |
| **Echocardiography** |  |  |  |  |  |  |
| Left Ventricular Hypertrophy |  |  |  |  |  |  |
| Yes | 27 (71.1) | 2.3 (0.8 – 6.5) | 0.1 |  | 2.2 (0.8 – 6.2) | 0.1 |
| No | 13 (52) | 1 |  |  |  |  |
| LVEF <40% |  |  |  |  |  |  |
| Yes | 28 (65.1) | 1.5 (0.6 – 4) | 0.4 |  | NA |  |
| No | 17 (54.8) | 1 |  |  |  |  |
| Left Atrial Dilation |  |  |  |  |  |  |
| Yes | 39 (67.2) | 3.4 (1.1 – 8.7) | 0.03 |  | 3.2 (1.04 – 10) | 0.03 |
| No | 6 (37.5) | 1 |  |  | 1 |  |
| Left Heart failure |  |  |  |  |  |  |
| Yes | 41 (63.1) | 2.1 (0.5 – 8.7) | 0.3 |  | NA |  |
| No | 4 (44.4) | 1 |  |  |  |  |
| **Etiology of Heart Failure** |  |  |  |  |  |  |
| Hypertensive Heart Disease |  |  |  |  |  |  |
| Yes | 27 (75) | 3.3 (1.2 – 8.9) | 0.02 |  | 3.1 (1.2 – 8.4) | 0.02 |
| No | 18 (47.4) | 1 |  |  | 1 |  |
| Ischemic Heart Disease |  |  |  |  |  |  |
| Yes | 0 (0) | NA |  |  | NA |  |
| No | 45 (61.6) | 1 |  |  |  |  |
| Dilated Cardiomyopathy |  |  |  |  |  |  |
| Yes | 5 (71.4) | 1.7 (0.3 – 9.3) | 0.6 |  | NA |  |
| No | 40 (59.7) | 1 |  |  |  |  |
| Cor pulmonale |  |  |  |  |  |  |
| Yes | 3 (37.5) | 0.3 (0.1 – 1.6) | 0.2 |  | 0.3 (0.1 – 1.6) | 0.2 |
| No | 42 (63.6) | 1 |  |  | 1 |  |
| Rheumatic Heart Disease |  |  |  |  |  |  |
| Yes | 4 (80) | 2.7 (0.3 – 25.8) | 0.04 |  | NA |  |
| No | 41 (59.4) | 1 |  |  |  |  |

aOR: adjusted Odds Ratio, BP: blood Pressure, CI: Confidence Interval, LVEF: Left Ventricular Ejection Fraction, NA: Not computed, NYHA: New York Heart Association
